# Persistent intrahepatic cholestasis secondary to multiple etiologies: a case report

**DOI:** 10.3389/fmed.2026.1830875

**Published:** 2026-06-25

**Authors:** Ran Wang, Yiyang Shang, Cai’e Wang, Yanhong Gao, Wenxiu Zhang, Fangbo Gao, Yang Guo, Baocheng Deng, Wenwen Zhang, Xingshun Qi

**Affiliations:** 1 Department of Gastroenterology, General Hospital of Northern Theater Command, Shenyang, China; 2 Department of Pharmacy, The First Affiliated Hospital of Jinzhou Medical University, Jinzhou, China; 3 Department of Blood Purification, General Hospital of Northern Theater Command, Shenyang, China; 4 Department of Infectious Diseases, The First Affiliated Hospital, China Medical University, Shenyang, China; 5 Department of Nuclear Medicine, General Hospital of Northern Theater Command, Shenyang, China

**Keywords:** adult-onset Still’s disease, glucocorticoids, intrahepatic cholestasis, liver biopsy, sepsis, severe liver injury

## Abstract

Intrahepatic cholestasis may arise from multiple etiologies, but it is particularly challenging to identify its primary etiology, especially when several potential contributors overlap and the disease itself progresses over time. Herein, we report a 42-year-old man with adult-onset Still’s disease (AOSD) who developed persistent severe intrahepatic cholestasis. At early stage of this disease, intrahepatic cholestasis was attributed to severe liver injury secondary to AOSD and drug-induced liver injury. Subsequently, sepsis developed, which might be associated with the use of glucocorticoids and contamination of central venous catheter, and aggravated intrahepatic cholestasis. Finally, the patient’s condition was gradually improved after catheter removal and targeted antibiotic therapy. This case highlights the importance of etiological reassessment in the management of persistent intrahepatic cholestasis.

## 1 Introduction

Intrahepatic cholestasis is a common manifestation of liver injury and may result from multiple etiologies. Major causes of intrahepatic cholestasis include viral hepatitis, alcoholic liver disease, drug-induced liver injury (DILI), pregnancy-related cholestasis, and sepsis (1). Since the identification of its etiology can guide the selection of treatment strategies, timely etiological assessment and targeted therapy are critical for improving clinical outcomes (2). However, in complex cases, it is often challenging to establish the primary etiology of cholestasis (3), and the integration of clinical manifestations, laboratory and imaging findings, and histopathology is required when indicated (4). Furthermore, multiple etiologies of liver injury may coexist (5, 6). As the disease evolves, the relative contribution of different etiologies or risk factors may also shift (7, 8), which further complicates etiologic attribution and clinical management. Therefore, etiological reassessment should be considered in patients with persistent intrahepatic cholestasis.

Here, we report a complex case of persistent intrahepatic cholestasis in a patient who developed severe liver injury secondary to adult-onset Still’s disease (AOSD) flare and possible DILI, and then sepsis associated with the use of glucocorticoids and contamination of central venous catheter (CVC).

## 2 Case report

On August 26, 2021 (day 1), a 42-year-old man was admitted to his local hospital with a 2-week history of jaundice, scleral icterus, poor appetite, skin rash, and severe fatigue. He had been taking oral herbal medicine (Pudilan Xiaoyan Oral Liquid) intermittently for chronic cough for over 2 months. He had been diagnosed with AOSD in 2001 and intermittently treated with corticosteroids. His peak temperature was 40 °C. Laboratory tests showed alanine aminotransferase (ALT) 1117 U/L, aspartate aminotransferase (AST) 800 U/L, total bilirubin (TBIL) 318.5 μmol/L, direct bilirubin (DBIL) 251 μmol/L, alkaline phosphatase (ALP) 165 U/L, and international normalized ratio (INR) 1.59. The Roussel Uclaf Causality Assessment Method (RUCAM) score was 5, indicating a possible diagnosis of DILI. He received hepatoprotective treatment with intravenous glutathione at a dosage of 1.8 g once daily.

On day 3, laboratory tests demonstrated TBIL 336.3 μmol/L, INR 2.12, ALT 1230.5 U/L, AST 816 U/L, serum albumin (ALB) 28.9 g/L, and ammonia 57 μmol/L. After treatment with intravenous methylprednisolone at a dosage of 40 mg once daily, hepatoprotective agents, including glutathione and ursodeoxycholic acid, and plasma transfusion, his temperature gradually decreased, but liver dysfunction persisted.

On day 5, laboratory tests demonstrated TBIL 359.8 μmol/L, DBIL 321.8 μmol/L, INR 2.3, ALT 1647 U/L, AST 1016 U/L, ALP 212 U/L, gamma-glutamyl transpeptidase (GGT) 404 U/L, ALB 28 g/L, white blood cell (WBC) count 13.2 × 10^9^/L, platelet (PLT) count 70 × 10^9^/L, C-reactive protein (CRP) 7.67 mg/L, and ferritin 7569 ng/mL; hepatitis A, B, C, and E virus, Epstein-Barr virus, and cytomegalovirus were all negative; and antinuclear antibody (ANA) and rheumatoid factor (RF) were negative. His temperature was 36.8 °C. Abdominal ultrasound indicated that his liver, pancreas, and spleen were normal. Bone marrow biopsy showed thrombocytopenia, but hemophagocytosis was not observed. Based on these findings, he was considered to have an AOSD flare complicated by severe liver injury. Subsequently, he underwent therapeutic plasma exchange (TPE) every 1–2 days and received magnesium isoglycyrrhizinate, glutathione, and ademetionine via a CVC, together with oral methylprednisolone at a dosage of 40 mg/day.

**FIGURE 1:**
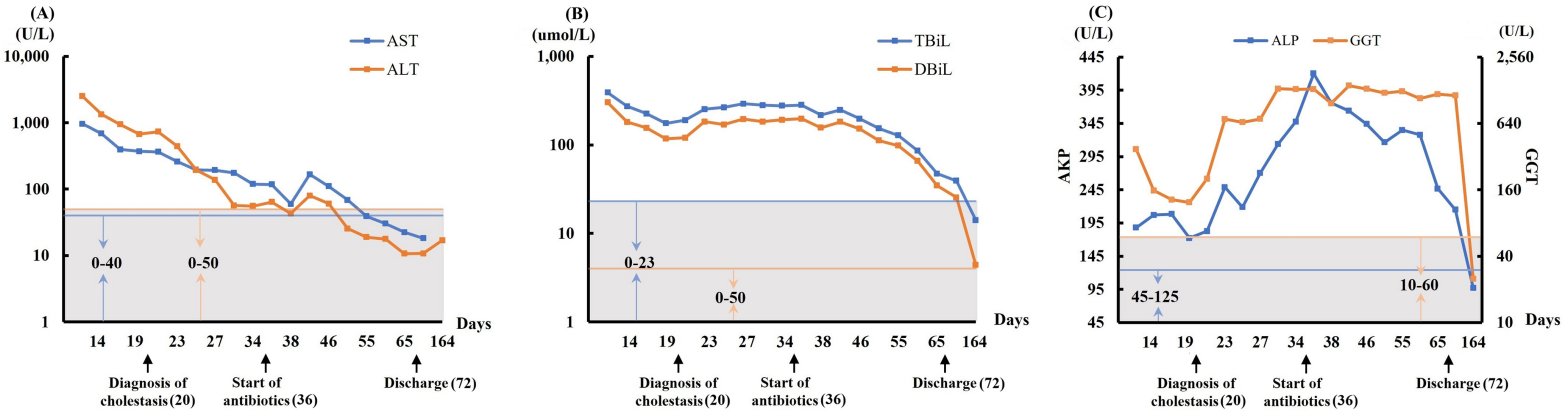
Changes of liver enzyme levels during hospitalization. **(A)** ALT and AST; **(B)** TBIL and DBIL; **(C)** ALP and GGT. ALT, alanine aminotransferase; AST, aspartate aminotransferase; TBIL, total bilirubin; DBIL, direct bilirubin; ALP, alkaline phosphatase, GGT, gamma-glutamyl transpeptidase.

**FIGURE 2:**
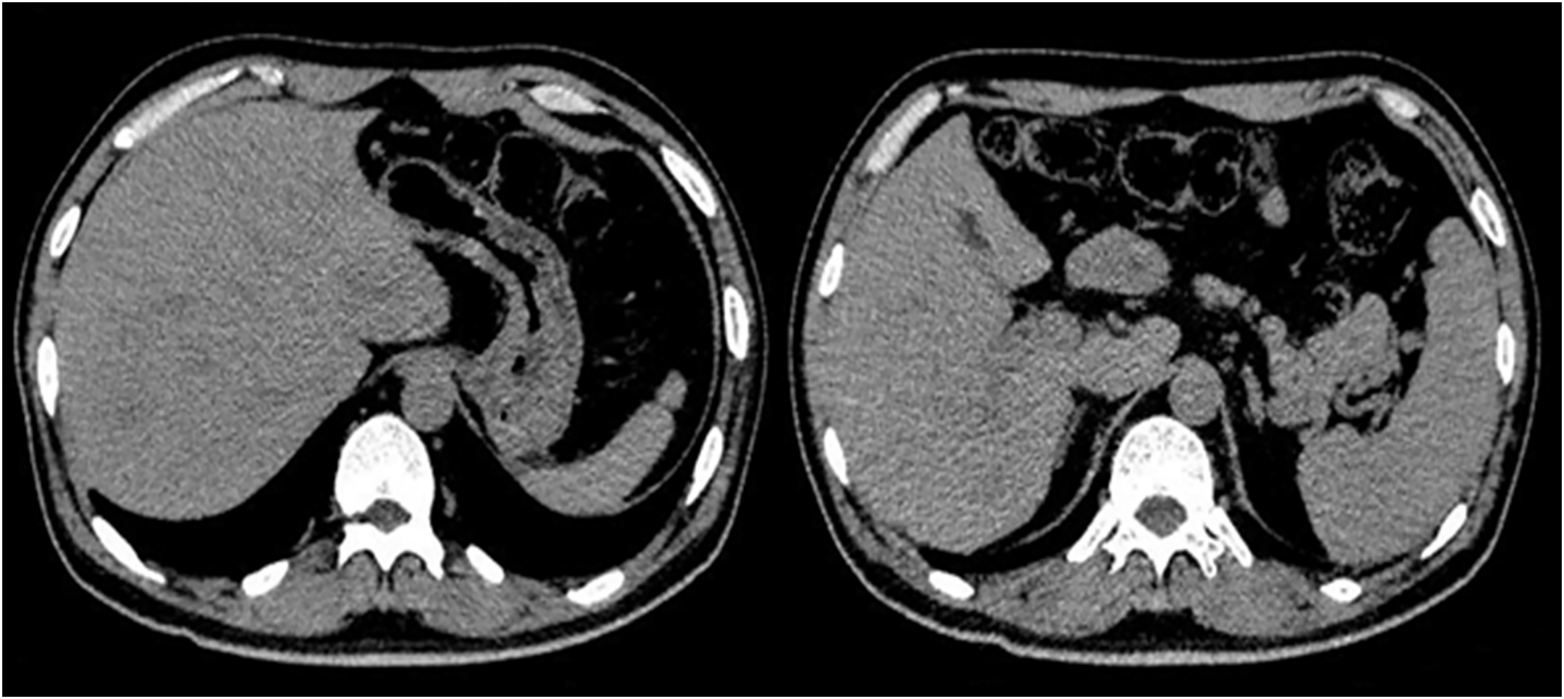
Plain abdominal CT.

**FIGURE 3:**
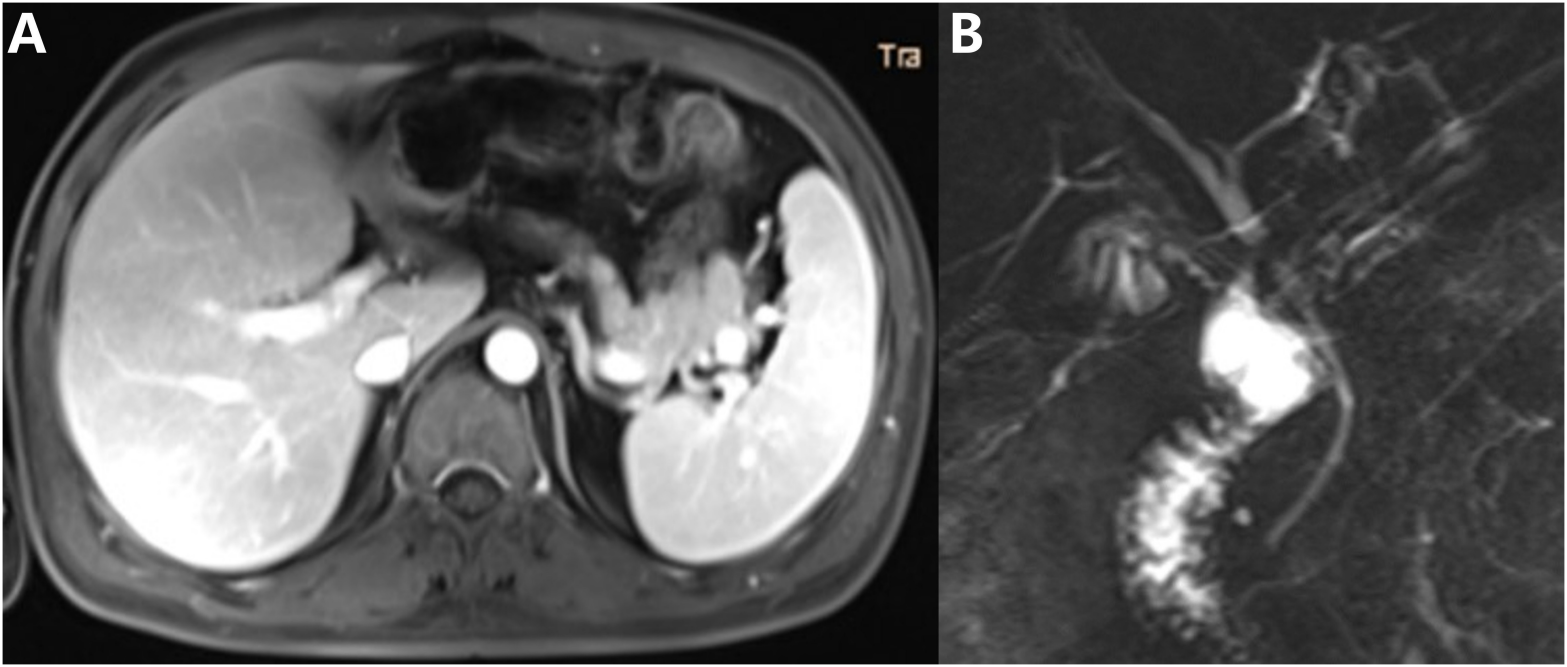
Contrast-enhanced MRI **(A)** and MRCP **(B)**.

On day 10, he was transferred to our hospital due to persistent liver dysfunction. Laboratory tests demonstrated TBIL 389.6 μmol/L, DBIL 301.4 μmol/L, ALT 2507.0 U/L, AST 961.0 U/L, ALP 188 U/L, GGT 375 U/L, ALB 29 g/L, INR 1.54, fibrinogen 2.34 g/L, D-Dimer 0.35 μg/ml, triglyceride 1.87 mmol/L, WBC 10.2 × 10^9^/L, PLT 105 × 10^9^/L, CRP 10.1 mg/L, and procalcitonin (PCT) 0.76 ng/ml. He underwent TPE on days 11, 13, 15, and 17. His ALT and AST levels significantly improved (Figure 1), whereas TBIL, GGT, and ALP levels continued to increase (Figure 1). Computed tomography (CT) scan performed on day 10 showed no biliary ductal dilatation (Figure 2). Magnetic resonance imaging (MRI) and magnetic resonance cholangiopancreatography (MRCP) performed on day 20 revealed a shrunken gallbladder (Figure 3). Extrahepatic biliary obstruction and malignancy were excluded based on CT and MRI/MRCP findings. No hepatic encephalopathy was noted. Further, he underwent TPE on days 22, 24, 26, and 27, as well as bilirubin adsorption on days 31, 33, and 36. After each TPE and bilirubin adsorption treatment, his bilirubin level decreased transiently but soon rose again (Figure 1). On day 31, CT-guided percutaneous liver biopsy was performed to further evaluate the cause of persistent cholestasis. His temperature remained normal until day 36. Due to persistent cholestasis, liver transplantation was recommended, but the patient declined.

At midnight on day 36, he suddenly developed chills, fever with a peak temperature of up to 40 °C, and arthralgia. At the same time, his heart rate was 112 beats/min, respiratory rate was 23 breaths/min, and blood pressure 70/40 mmHg. Arterial blood gas analysis showed pH 7.43, PaO_2_ 75 mmHg, PaCO_2_ 46 mmHg, lactic acid 2.0 mmol/L, FiO_2_ 29%, and SaO_2_ 95%, corresponding to a PaO_2_/FiO_2_ ratio of 293. In the setting of suspected infection, he had new-onset hypotension and respiratory compromise, and the overall Sequential Organ Assessment (SOFA) score was 4 (9). These findings fulfilled the Sepsis-3 criteria for sepsis (9). Septic shock was then considered because of hypotension. Blood culture was obtained immediately, and he received fluid resuscitation, antipyretic therapy, and intravenous ceftriaxone 2 g/day. After urgent multidisciplinary consultation on day 37, antibiotic therapy was changed to intravenous imipenem/cilastatin at a dosage of 500 mg every 8 h. Then, his fever subsided, and his laboratory tests showed WBC 10.0 × 10^9^/L, CRP 53.81 mg/L, and PCT 7.8 ng/ml. Given the potential risk of catheter-related bloodstream infection, his CVC was removed, and catheter tip culture was performed.

**FIGURE 4:**
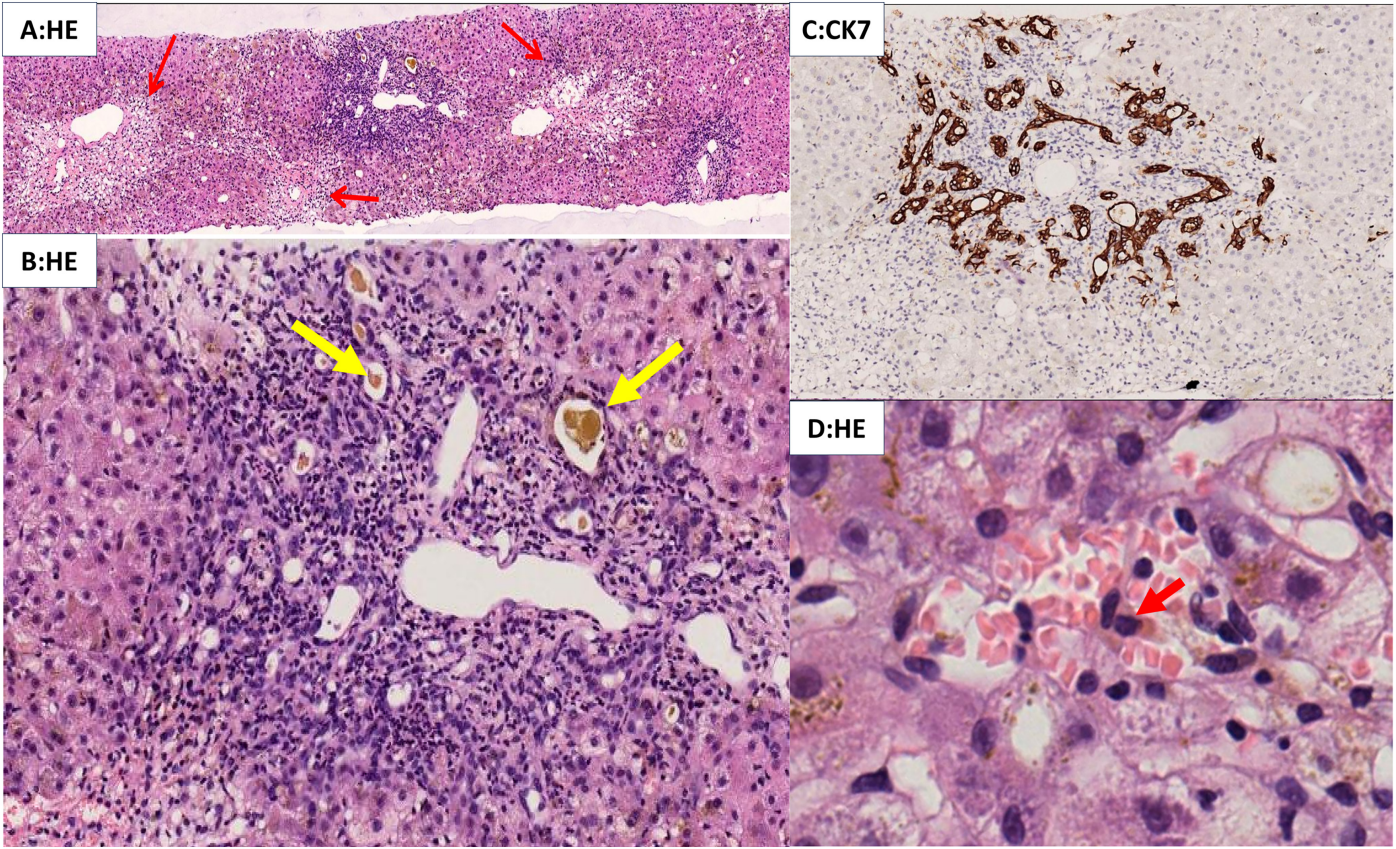
Liver histology [hematoxylin and eosin, (HE)]. **(A)** Hepatocytes; **(B,C)** HE and CK7, portal tracts show multiple dilated bile ductules in the periportal stroma, with bile plugs in some of the lumina (yellow arrows). **(D)** CK7, Erythrophagocytosis by sinusoidal Kupffer cells is observed.

On day 38, liver histology showed lobular fusion necrosis, intrahepatic cholestasis, and hemophagocytosis (Figure 4). Notably, the presence of bile plugs within dilated periportal ductules was histologically compatible with sepsis-associated cholestatic injury. Methylprednisolone was gradually tapered by 5 mg per week from day 38 to day 94.

On day 39, he responded well to imipenem/cilastatin. Blood culture results showed *Staphylococcus aureus*. Catheter tip culture also showed the presence of *Staphylococcus aureus* (>15 CFU). Therefore, he continued intravenous imipenem/cilastatin at a dosage of 500 mg every 8 h. On day 44, his temperature remained normal, and imipenem/cilastatin was changed to intravenous cefazolin at a dosage of 2 g every 6 h. On day 53, he had no fever or chills, and laboratory tests showed WBC 5.9 × 10^9^/L and CRP 43.79 mg/L. Cefazolin was then discontinued. TBIL level gradually decreased (Figure 1).

On day 68, his liver-related biomarkers had normalized (Table 1). He was discharged on day 72. On April 24, 2023, his liver function remained normal. Written informed consent for publication was obtained from the patient.

**TABLE 1:** Patient’s laboratory test results.

Day	TBiL (umol/L)	DBiL (umol/L)	ALT (U/L)	AST (U/L)	ALP (U/L)	GGT (U/L)	PT (s)	INR	WBC (10∧9/L)	RBC (10∧12/L)	Hb (g/L)	PLT (10∧9/L)	CRP (mg/L)	PCT (ng/ml)	FIB (g/L)	D-dimer (μg/ml)	IL-6 (pg/ml)	Ferritin (ng/ml)	Temperature (°C)	HR (bpm)	RR (brpm)	BP (mm/Hg)
1	318.5	251	1117	800	145	258	21.4	1.67	13.2	5.35	149	70	7.67	0.769	2.34	0.35	1.8	7569	40	86	17	125/77
3	336.3	262.6	1230.5	816	153	302	27.1	2.12											36.8			
5	359.8	321.8	1647	1016	212	404	30.9	2.41														
10	389.6	301.4	2507	961	188	375	19.7	1.54	10.2	5.47	158	105	10.1						36.7	79	15	134/82
13	273.3	180.7	1335.1	686.64	206.98	157.92	15.8	1.23	10.3	4.45	133	111	3.29		2.08	0.43	28.54	8488	36.5	90	14	133/80
15	225.3	155.2	943.56	395.11	208.31	130.37	16	1.25	10.4	4.42	126	131			1.92	0.37			36.6	70	16	131/86
18	175.4	117.5	673.67	370.07	172.23	123.09	15.3	1.18	11.6	4.16	118	119			1.69	0.25			37.0	94	16	110/74
19	189.9	120.5	731.87	365.16	182.69	201.34	14.6	1.12	12.5	4.25	122	124	2.64		1.59	0.28			36.5	71	14	123/77
22	253.3	183.3	443.17	259.42	249.02	700.1	16	1.25	11	4.24	125	109			2.16	0.33			36.9	86	16	114/73
24	265.7	169.4	194.68	195.01	218.94	656.29	15.3	1.18	9.2	4.09	124	89	3.7		1.94	0.27		3811	36.5	79	14	139/80
26	291.8	195.9	137.66	192.4	270.45	702.62	14.3	1.09	8.1	4.24	124	100			2.22	0.25			36.9	84	14	114/70
31	280.1	183.2	56.52	175.55	313.62	1321.7	14.6	1.12	6.8	4.21	127	97	7.55		2.68	0.59			36.5	84	14	128/76
33	276.9	191.7	55.54	118.87	347.71	1308.3	14.4	1.1	8.1	4.23	128	95	22.86		3.34	0.42		8787	37.0	70	17	101/60
36	281.6	197.6	64.06	117.42	420.11	1313.4	16.4		10	3.64	113	114	53.81	7.8	3.07	0.57			40	112	23	70/40
37	216.6	156.3	42.8	59.774	375.86	974.07	13		7.4	3.57	110	132	74.51	3.3	3.19	0.36			38.1	98	20	93/65
41	247.6	181.9	80.02	167.04	363.85	1410.1	13.9	1.05	8.7	3.33	107	124	20.96	0.9	3.21	0.45	17.9		36.6	73	17	129/83
45	197.1	152.2	60.22	110.64	344.18	1318.9	14.2		10.6	3.08	104	122	33.61	0.58	2.54	0.62	23.49		36.9	76	16	126/78
50	153.5	111.9	25.41	68.54	316.62	1208.8	13.6	1.02	6	2.61	92	110	28.57	0.41	2.94	1.06		4513	36.7	92	15	129/81
54	127.7	98.2	18.87	38.98	334.91	1250.2			7.5	2.77	100	115	34.51	0.1	2.38	0.64			36.5	78	16	111/73
58	85.6	65.7	17.69	30.16	327.68	1081.1			6	2.41	109	108	22.34						36.7	95	16	126/78
64	47.4	34.7	10.67	22.49	246.78	1179.7	13.5		8.1	1.94	112	123	6.08						36.5	94	14	123/78
68	39.3	25.5	10.74	18.2	214.97	1148.8			7.5	2.09	107	132	5.84						36.7	87	17	115/69
244	14.1	4.40	17		97	25																
607	4.9	1.8	25.86	21.07	85.76	85.57																

ALT, alanine aminotransferase; ALP, alkaline phosphatase; AST, aspartate aminotransferase; BP, blood pressure; CRP, C-reactive protein; DBiL, direct bilirubin; FIB, fibrinogen; GGT, gamma-glutamyltranspeptidase; Hb, hemoglobin; HR, heart rate; IL-6, interleukin-6; INR, international normalized ratio; PCT, procalcitonin; PLT, platelet; PT, prothrombin time; RBC, red blood cell; RR, respiratory rate; TBiL, total bilirubin; WBC, white blood cell.

## 3 Discussion

This is a complex case of persistent intrahepatic cholestasis due to multiple insults with a long disease course. His initial severe liver injury with cholestasis was multifactorial, occurring in the setting of AOSD flare combined with possible DILI. Intrahepatic cholestasis remained in spite of TPE and hepatoprotective therapy. Later, the patient developed septic shock which was supported by the positive result of both blood and catheter tip cultures. Following catheter removal and targeted antibiotic therapy, the patient’s clinical symptoms resolved and laboratory parameters improved. In addition, liver histological findings were compatible with severe cholestatic injury associated with sepsis. This case highlights the need for repeated etiological assessment in patients with persistent intrahepatic cholestasis. In patients with immune-mediated diseases receiving corticosteroids, the clinical manifestations of infection may be attenuated, potentially delaying the recognition of sepsis as a contributor to persistent intrahepatic cholestasis.

At early stage of liver injury, several potential etiologies may have been involved. First, AOSD-associated liver injury has been described in several case reports (10–13). Our patient was diagnosed with AOSD flare based on a history of AOSD, recurrent fever, rash, marked hyperferritinemia, leukocytosis, negative ANA and RF results, and fulfillment of Yamaguchi’s criteria (14). Ott et al. reported a patient with AOSD who was successfully treated with glucocorticoids, but developed liver failure (15). Second, DILI was another potential etiology because the patient had taken herbal medicine intermittently for 2 months before the onset of liver injury. However, the RUCAM score was only 5, indicating a possible causal relationship between herbal medicine and liver injury (16). Notably, his initial liver injury showed hepatocellular pattern, with markedly elevated ALT and AST levels. As the disease course evolved, the pattern of liver injury shifted from hepatocellular to cholestatic. This is consistent with previous findings that the pattern of DILI may change over time (8, 17, 18).

Macrophage activation syndrome (MAS) was also considered, given the underlying AOSD (19), marked hyperferritinemia, thrombocytopenia, and hemophagocytosis on liver histology (20). However, the available evidence was insufficient to support a definite diagnosis of MAS. In the early phase, although ferritin was markedly elevated, the patient didn’t have any typical clinical and laboratory profile of MAS, including persistent fever, hypofibrinogenemia, hypertriglyceridemia, or significant D-dimer elevation. Bone marrow examination also showed no hemophagocytosis. In the late phase, hemophagocytosis identified on liver histology alone was not specific for a diagnosis of MAS, and may also occur in the setting of sepsis-related immune activation (21). Therefore, MAS could not be completely excluded, but was considered less likely to be the primary cause of persistent cholestasis.

Intrahepatic cholestasis persisted, although liver injury partially resolved after treatment in our patient. Persistent cholestasis may occur even after the triggering factor has been removed. Several case reports have documented persistent cholestasis following viral hepatitis (22, 23), DILI (24), and Coronavirus disease 2019 (25). van Dijk et al. defined persistent hepatocellular secretory failure (PHSF) as follows: (1) serum bilirubin > 255 μmol/L (>15 mg/dL); (2) persistent or further increasing serum bilirubin levels for >1 week after removal of the underlying trigger; (3) exclusion of obstructive cholestasis by imaging; and (4) no evidence of pre-existing liver disease (26). Accordingly, PHSF may be considered in our case, where liver biochemical parameters partially improved after glucocorticoid therapy, but intrahepatic cholestasis persisted (Figure 1). However, the patient’s condition improved after the removal of the CVC and antibiotic treatment, suggesting that sepsis played an important role in the late course of the disease. In addition, genetic testing was not performed in our case, so it was not possible to further assess whether host susceptibility factors contributed to PHSF.

Sepsis played an important role in the late phase of the disease course in our case. Major risk factors for sepsis included glucocorticoid therapy for AOSD and CVC placement. Glucocorticoid exposure may predispose patients to infection through immunosuppression and may also mask its clinical manifestations, thereby delaying diagnosis and intervention (27). In addition, prolonged CVC dwell time increased the likelihood of catheter-related bloodstream infection. Liver histology showed dilated periportal ductules containing bile plugs, representing a characteristic pathological manifestation of sepsis (28). Histology findings combined with a decrease in TBIL level after catheter removal and antibiotic treatment supported sepsis-associated cholestasis. Therefore, we considered that sepsis aggravated the pre-existing cholestatic liver injury in our case.

The underlying pathophysiology of sepsis-associated cholestasis is as follows: first, hepatic Kupffer cells clear bacteria and endotoxins from the portal circulation to modulate systemic inflammation. In patients with sepsis, circulating bacterial endotoxins activate Kupffer cells and release a massive cascade of soluble mediators, including proinflammatory cytokines, such as TNF-α, IL-1, IL-6, and IL-12, reactive oxygen species, and nitric oxide (1, 29, 30). These mediators collectively induce sinusoidal endothelial damage, promote microthrombi formation, and downregulate critical hepatobiliary transporters, ultimately culminating in intrahepatic cholestasis. Second, in some cases of severe sepsis, especially septic shock, systemic hypotension may induce biliary ischemia, and then cause a distinct phenotype of progressive sclerosing cholangitis (1). This mechanism may be important for explaining the disease course of our case.

Our case has several limitations. First, some laboratory data, such as PCT, were missing or unavailable before the referral to our department, compromising a more comprehensive assessment of disease progression in this case. Second, based on the RUCAM score, only a possible diagnosis of DILI was made, and liver histology was missing at early stage of the disease.

## 4 Conclusion

Based on this case, we emphasize the importance of etiological assessment in patients with severe intrahepatic cholestasis during a long disease course. In addition, clinicians should be aware that sepsis, as a potential etiology of severe intrahepatic cholestasis, may be masked in patients receiving glucocorticoid therapy.

## Data Availability

The original contributions presented in this study are included in this article/Supplementary material, further inquiries can be directed to the corresponding author.
